# The effect of acupuncture on the expression of inflammatory factors TNF-α, IL-6,IL-1 and CRP in cerebral infarction: A protocol of systematic review and meta-analysis

**DOI:** 10.1097/MD.0000000000015408

**Published:** 2019-06-14

**Authors:** Yuru Chen, Wei Huang, Zunjiang Li, Yunbiao Duan, Zhaoxiong Liang, Hong Zhou, Chuyue Tang

**Affiliations:** a The Second Clinical College of Guangzhou University of Chinese Medicine, Guangzhou; b Jiangmen City Hospital of Chinese Medicine, Jiangmen; c Guangdong Provincial Hospital of Chinese Medicine, Guangzhou, Guangdong, China.

**Keywords:** acupuncture, cerebral infarction, inflammatory factors, meta-analysis, systematic review

## Abstract

**Background:**

The mechanisms of acupuncture on the treatment of cerebral infarction remain unclear, the aim of the present study was to provides a protocol of systematic review and meta-analysis, with which we will collect clinical evidence to verify whether acupuncture will have an effect on reducing the levels of tumor necrosis factor α (TNF-α), C-reactive protein (CRP), interleukin-1 (IL-1), and interleukin (IL-6) after cerebral infarction based on evidence-based studies.

**Methods:**

Included studies will be retrieved according to inclusion and exclusion criteria from 5 English databases (the MEDLINE via PubMed, the Cochrane Library, Embase, the Web of Science, and Ovid database), and 4 Chinese databases (China Science and Technology Journal Database (VIP), Chinese Biomedical Literature Database (CBM), Wan-fang Database, China National Knowledge Infrastructure (CNKI)) from October 1990 to October 2017. The inflammatory factor levels of TNF-α and IL-1,IL-6,CRP will be marked as major outcomes. We will use RevMan V.5.3 software to calculate the data synthesis and will conduct meta-analysis based on the collected data.

**Results:**

The inflammatory factor levels of TNF-α and IL-1,IL-6,CRP, mortality and adverse effects will be measured and comprehensively assessed to evaluate the adjunctive effect of XBP on CHF from this systematic review and meta-analysis with current clinical evidence.

**Conclusion:**

The systematic review and meta-analysis will assess the effect of acupuncture on the expression of inflammatory factors TNF-α, IL-6, IL-1 and CRP in cerebral infarction with up-to-date clinical evidence.

**PROSPERO registration number:**

PROSPERO CRD42017078583.

## 1 Introduction

Cerebral infarction has been one of common types of stroke in China, and ischemia stroke has been the second largest cause of death for Chinese residents, ranking third in the developed world.^[1–4]^ Cerebral infarction would results in complex complications and dysfunctions because of the perfusion restored in ischemic area following cerebral ischemia.^[1,2]^ Sudden interruption flow of blood in local or extensive brain tissue activates the expression of inflammatory cytokines such as TNF-α, IL-1, CRP and IL-6 etc., which aggregates neutrophils, monocytes and T cells into the brain injury tissue, and enhances the inflammatory damage.^[4,5]^ Many studies have shown that inflammatory factors act as important factors in influencing the prognosis, pathophysiological and pathological process of ischemic infarction. Controlling the progression of inflammation could be conducive to reducing the adverse consequences and have a positive effect on its prognosis.^[6–9]^

Acupuncture has been treated as a medical therapy and has integrated with modern medical more closely in recent years.^[10]^ It also plays as a safe clinical treatment widely adopted by the therapists and the patients in Western countries.^[11,12]^ Acupuncture treatment includes body needles, electro-acupuncture, scalp-acupuncture, ear needles, and other types or other forms.^[13]^ Acupuncture is of great importance in regulating physiological changes, but some of the specific mechanisms of the treatment are still unclear.^[14–17]^

Many experimental studies have explored that acupuncture can significantly minimize the adverse impacts of brain cerebral, through reducing expression on inflammatory factors and controlling the harmful effect during the process of cerebral ischemia/reperfusion after cerebral infarction.^[18–21]^ In clinical studies, it was also reported that acupuncture was beneficial to the recovery by reducing the level of inflammatory factors, while these studies has not assessed the clear or consistent results of acupuncture intervention. Further certifications for standard treatments are still remained.^[22]^ In a recent multicenter randomized trial, it is suggested that acupuncture had an additional effect on early comprehensive rehabilitation of patients with mild to moderate acute ischemic stroke, but not brought out whether acupuncture was beneficial to the recovery of cerebral infarction by reducing the expression of inflammatory factors.^[23]^ Until now, literature search has found that there is still no meta-analysis on the efficacy of acupuncture on the inflammatory factors.^[24]^

Thus, this present study is performed to verify the efficacy and safety of acupuncture for cerebral infarction with clinical evidence-based studies, and systematically elaborate the specific role of acupuncture in reducing the level of inflammatory factors and whether it was facilitated to recovery after brain ischemia, with the purpose of summarizing the published clinical research and offering recommendations for future research or clinical treatments, which also benefited our next research and would provide theories or guidance for our further research or other studies involving the mechanism of acupuncture.

## 2 Method

### 2.1 Inclusion criteria for study selection according PICOs criteria

The eligibility criteria were strictly detailed with the PICOS framework,^[25]^ which was showed as follows: Population: ischemia stroke in adults in accordance with the Guidelines on the Diagnosis, inpatients and outpatients, animal studies excluded; Intervention: All kinds of acupuncture method with relate acupuncture points either auricular or body; Control: sham acupuncture or treatment as usual or other conventional treatment; Outcomes: the inflammatory factor levels of TNF-α and IL-1,IL-6,CRP; Study design: random control studies, restricted to English and Chinese language publications.

### 2.2 Data search method

There independent reviewers (LZJ, DYB, DBH) searched from 1990 to 2017 in English or Chinese with no publication restrictions through MEDLINE via PubMed, the Cochrane Library, Embase, the Web of Science, and Chinese language databases including China Science and Technology Journal Database, Chinese Biomedical Literature Database, Wan-fang Database, and China National Knowledge Infrastructure were also searched in the same period. Standards-compliant conference articles and Doctoral or master theses were also included. The keywords used in the literature search were shown in Table 1. Our search topics are composed of P+I+C+O+S, P+I+C+O and P+I+O in order to ensure the search rate of literature search. Some nonpublished paper or detailed data will be obtained by emailing corresponding authors.

**Table 1:**
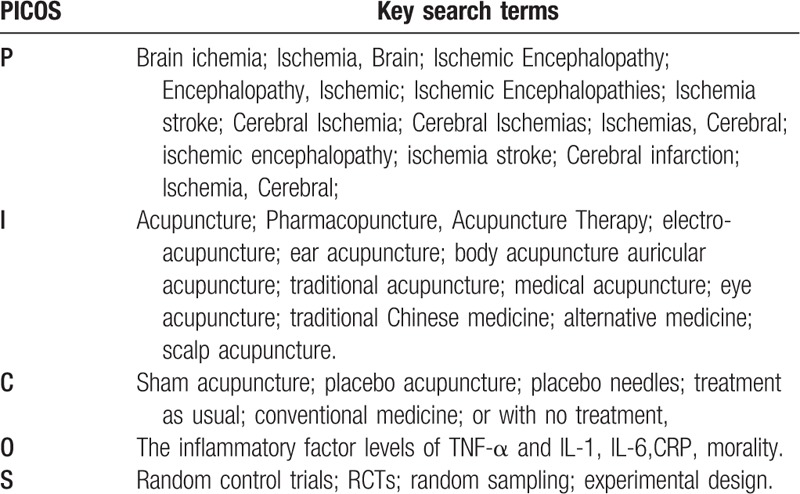
Key terms (or nearest appropriate equivalent) in search method.

### 2.3 Study selection

**Figure 1:**
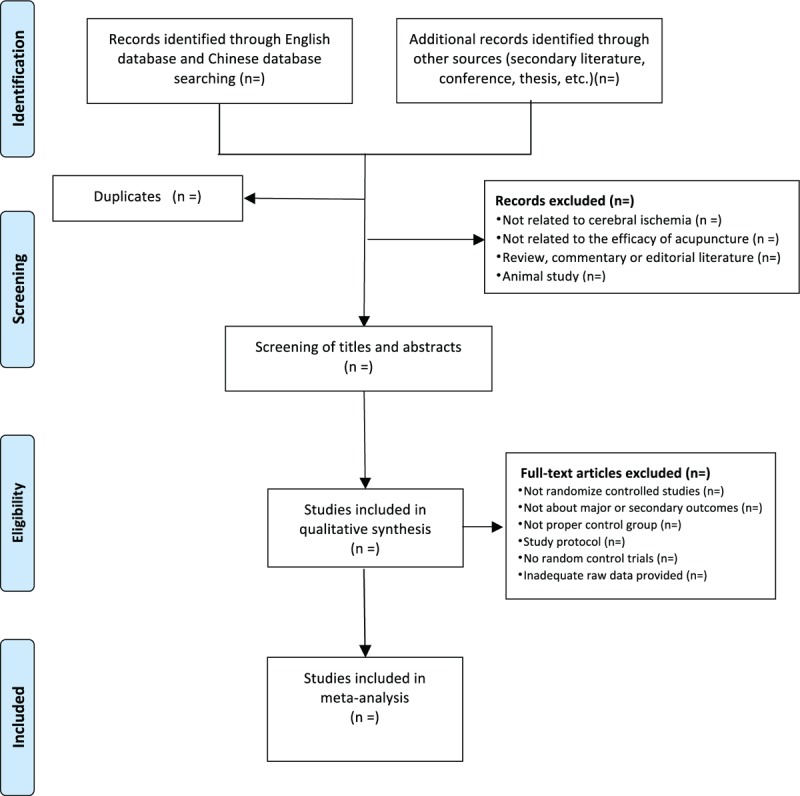
PRISMA flowchart of study selection process in the systematic review.

All the random control studies will be screened by three independent researchers (CYR, LZX, and HW) according to titles and abstracts firstly before full texts are further evaluated. Duplicate literature will be removed. Studies removed after full text review will be recorded with specific exclusion reason. All the RCTs will be compared with control groups in the way of all types of sham acupuncture or conventional medicine. Any selected study will fulfill the requirements of inclusion criteria.^[26]^ Two experienced authors (TCY and ZH) will translate the full Chinese reports, and his translations will be clarified by a third author (HW) for further evaluation. Disagreements will be resolved by discussion for consensus among the reviewers. The selection process of eligible papers is shown in a Preferred Reporting Items for Systematic Review and Meta-analysis (PRISMA) flow diagram^[27]^ (Fig. 1).

### 2.4 Risk of bias assessment

Our review will assess bias of the studies with the Cochrane Risk of Bias tool^[26]^ consisting of following items: random sequence generation and allocation concealment (selection bias); blinding of participants and personnel (performance bias); blinding of outcome assessment (detection bias); incomplete outcome data (attrition bias); selective reporting (reporting bias); and other potential sources of bias. Methodological quality was evaluated by using the Collaboration Review Manager software (Version 5.3, Copenhagen: Nordic Cochrane Center, Cochrane Collaboration, 2014). All the items were evaluated by 3 reviewers (DYB, DBH, and TCY) independently, any divergence were resolved by discussion or the third reviewer (CYR).

### 2.5 Data extraction and management

#### 2.5.1 Data extraction

The same 3 reviewers (CYR, LZX, and HW) will extract data independently using the special forms base on all the identified studies (Table 2, Supplemented in end of the text). Data extraction will include study characteristics, population characteristics, average age, sample size, duration, details of the treatment and control group, details of the outcomes and adverse events. All the data and disagreement between the 2 reviewers will be evaluated by a third reviewer (CYR) who served as an arbiter for a final decision throughout the entire procedure.

**Table 2:**
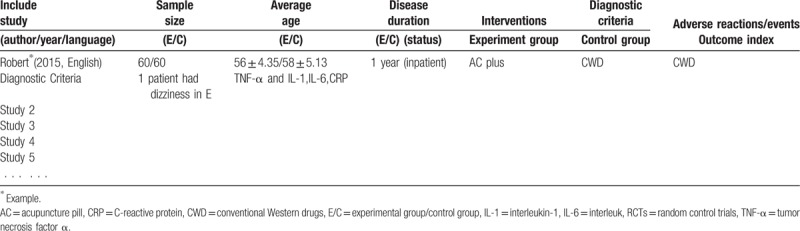
Characteristics of included RCTs investigating the effect of acupuncture on cerebral ischemia.

#### 2.5.2 Dealing with missing data

Any insufficiency of the above-mentioned data will be supplemented by contacting the corresponding authors using e-mail or telephone. Potential impact of unavailable data will be added in the discussion part for further evaluation on the results.

#### 2.5.3 Quantitative synthesis

For continuous data, when the change of the mean (Mean_change_) and standard deviation (SD_change_) of inflammation levels in pretreatment and post-treatment were provided, we compared the change from baseline to endpoint; When the mean difference (Mean_change_) were provided without providing mean difference standard error (SD_change_), we calculate the SD_change_ with the follow formula.^[28]^ If there are 3 groups, we will compare the data of conventional therapy group with the data of the XBP plus conventional therapy group. Only the last available time point reported on the outcome if the study has different time points throughout the intervention.  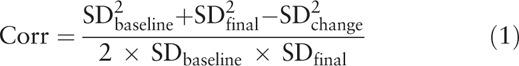



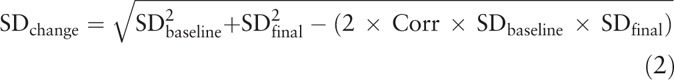


#### 2.5.4 Selection of effects model

The standard mean difference with 95% confidence interval (CI) will be used to evaluate the continuous data, while dichotomous outcomes will be measured with the rate ratio (RR) with 95% CIs. If *I*^2^ < 50, we will perform the fixed-effects model to calculate the RR and mean difference, but we will choose the random-effects model if *I*^2^ ≥ 50.

### 2.6 Analysis of the data

#### 2.6.1 Assessment of heterogeneity

The chi-squared test will be applied to evaluate the heterogeneity with the cut-off value of *I*^2^ = 50 according to the guideline of Cochrane Handbook. If *I*^2^ > 50%, trials will be considered with significant heterogeneity, and subgroup analysis will be necessarily performed to assess the potential heterogeneity sources.

#### 2.6.2 Subgroup analysis

If it is considered with high heterogeneity, subgroup analysis will be performed to reduce the heterogeneity and ensure the accuracy of results including onset time and duration of illness, age and number of samples, etc., to investigate which variables best predict the enhancement effect of acupuncture.

#### 2.6.3 Sensitivity analysis

Sensitivity analysis will be also applied to evaluate the robustness and reliability of the combined results of included studies. Methodological quality, heterogeneity, studies quality (according to the 4 levers: high, moderate, low, or very low) and sample characteristic will be considered.

#### 2.6.4 Assessment of reporting biases

We will conduct analysis of Egger's publication bias plot and Begg funnel plot with pseudo 95% confidence limits to determine the publication bias in all the literature with sufficient studies (more than 10 trials).

#### 2.6.5 Test sequential experiment

In order to illustrate and confirm the credibility of our results, we performed a sample size analysis by trial sequential analysis (TSA), which aims to rule out the possibility of false positives.^[29]^

### 2.7 Ethics approval

All the data will be extracted from the published studies through database, which is not directly related to patient data, thus not ethical approval is require. The findings of this systematic review will provide implication of the enhancement effectiveness of XBW for CHF. The systematic review will be disseminated in a peer-reviewed journal and published at conference presentations, which will be served as the guidance for a multi-center random control trial in our further research.

## 3 Discussion

Cerebral ischemia is the most common cause of disability, the second commonest cause of dementia and the fourth commonest cause of death in the developed world.^[30]^ In the United States of America, every year about 795,000 people suffer from a new or recurrent stroke. In Switzerland, stroke incidence has been estimated at 150/100,000 inhabitants,^[31]^ causing a large amount of direct and indirect costs.^[32]^ In China, the number of disability and deaths caused by cerebrovascular diseases, especially cerebral infarction, has exceeded 5 million per year, 50% to 70% of the patients survive after treatment have different degrees of disability.^[33,34]^ Acupuncture is a safe and effect method applied in the treatment of cerebral ischemia, and has been widely adopted by the therapists and the patients in Western countries.^[11,12]^ It is also treated as an alternative therapy for cerebral ischemia because of its clinical efficacy.^[10]^

Many experimental and clinical studies have explored that acupuncture can significantly minimize the adverse impacts of brain cerebral, through reducing expression on inflammatory factors and controlling the harmful effect during the process of cerebral ischemia/reperfusion after cerebral infarction.^[18–21]^ But the efficacy of acupuncture in the treatment of cerebral ischemia is still uncertain. Therefore, it is necessary to conduct high-quality systematic evaluation and meta-analysis on it. Our standardized methods will provide an objective evidence-based review for acupuncture in the treatment of cerebral ischemia. We will use high-quality clinical evidence to demonstrate whether acupuncture has the effect of improving the clinical symptoms, reducing the inflammatory factor levels of TNF-α and IL-1,IL-6,CRP and reducing the occurrence of adverse events on the basis of conventional treatment. In addition, whether acupuncture combined with conventional treatment can be more effectively to improve the primary and secondary outcomes will be verified. However, there are limitations in this systematic review that may affect the drawn conclusion. First, the included trials are mainly restricted to the published results, which may run risk of publishing bias. Second, different times of onset and types of cerebral ischemia may lead to heterogeneity in our results in terms of that subgroup analysis may be unavailable if the quantities of included studies are not enough. Thus, in order to avoid these, we will conduct this analysis with more studies and more extra unpublished results as we can as possible.

## Author contributions

Hong Zhou and Yuru Chen designed and evaluated the review, Chuyue Tang, Wei Huang and Yunbiao Duan selected the trials by search. Yuru Chen and Zhaoxiong Liang translated and clarified the full Chinese reports. Zunjiang Li, Yuru Chen and Chuyue Tang evaluated the all process conducted in the analysis and extraction of the data. All authors contributed to the evaluation of review and analysis and approved the final version submitted for publication.

**Conceptualization:** Yuru Chen, Hong Zhou, Chuyue Tang.

**Data curation:** Yuru Chen, Zunjiang Li, Yunbiao Duan, Zhaoxiong Liang, Chuyue Tang.

**Formal analysis:** Zunjiang Li, Yunbiao Duan, Chuyue Tang.

**Investigation:** Zhaoxiong Liang.

**Methodology:** Wei Huang, Zhaoxiong Liang.

**Supervision:** Hong Zhou.

**Validation:** Yuru Chen.

**Writing – original draft:** Yuru Chen, Zunjiang Li, Wei Huang, Chuyue Tang.

**Writing – review & editing:** Yuru Chen, Wei Huang, Yunbiao Duan, Hong Zhou.

Chuyue Tang orcid: 0000-0003-0605-3333.
